# Integrating Signals from Sperm Methylome Analysis and Genome-Wide Association Study for a Better Understanding of Male Fertility in Cattle

**DOI:** 10.3390/epigenomes3020010

**Published:** 2019-05-16

**Authors:** Lingzhao Fang, Yang Zhou, Shuli Liu, Jicai Jiang, Derek M. Bickhart, Daniel J. Null, Bingjie Li, Steven G. Schroeder, Benjamin D. Rosen, John B. Cole, Curtis P. Van Tassell, Li Ma, George E. Liu

**Affiliations:** 1 Animal Genomics and Improvement Laboratory, BARC, Agricultural Research Service, USDA, Beltsville, MD 20705, USA; 2 Department of Animal and Avian Sciences, University of Maryland, College Park, MD 20742, USA; 3 Key Laboratory of Agricultural Animal Genetics, Breeding and Reproduction, Education Ministry of China, Huazhong Agricultural University, Wuhan 430070, China; 4 Key Laboratory of Animal Genetics, Breeding and Reproduction, Ministry of Agriculture & National Engineering Laboratory for Animal Breeding, College of Animal Science and Technology, China Agricultural University, Beijing 100193, China; 5 Dairy Forage Research Center, Agricultural Research Service, USDA, Madison, WI 53718, USA

**Keywords:** sperm DNA methylation, male fertility, large-scale GWAS, cattle complex traits

## Abstract

Decreased male fertility is a big concern in both human society and the livestock industry. Sperm DNA methylation is commonly believed to be associated with male fertility. However, due to the lack of accurate male fertility records (i.e., limited mating times), few studies have investigated the comprehensive impacts of sperm DNA methylation on male fertility in mammals. In this study, we generated 10 sperm DNA methylomes and performed a preliminary correlation analysis between signals from sperm DNA methylation and signals from large-scale (*n* = 27,214) genome-wide association studies (GWAS) of 35 complex traits (including 12 male fertility-related traits). We detected genomic regions, which experienced DNA methylation alterations in sperm and were associated with aging and extreme fertility phenotypes (e.g., sire-conception rate or SCR). In dynamic hypomethylated regions (HMRs) and partially methylated domains (PMDs), we found genes (e.g., *HOX* gene clusters and microRNAs) that were involved in the embryonic development. We demonstrated that genomic regions, which gained rather than lost methylations during aging, and in animals with low SCR were significantly and selectively enriched for GWAS signals of male fertility traits. Our study discovered 16 genes as the potential candidate markers for male fertility, including *SAMD5* and *PDE5A*. Collectively, this initial effort supported a hypothesis that sperm DNA methylation may contribute to male fertility in cattle and revealed the usefulness of functional annotations in enhancing biological interpretation and genomic prediction for complex traits and diseases.

## 1. Introduction

Decreased male fertility is a big concern in both human society and the livestock industry [1,2]. In mammals, global reprogramming of DNA methylation patterns happens twice during development: Once during early embryogenesis and once during germ cell development. Because of its crucial role in the normal embryonic development through regulating gene expression, sperm DNA methylation is often believed to be associated with male fertility [3,4,5,6,7,8,9]. Hammoud et al. (2009) demonstrated the promoters of developmental genes (e.g., microRNA clusters and HOX gene clusters) were generally hypomethylated in the human sperm, and the retained nucleosomes in sperm were significantly enriched at these genes, revealing important contributions of sperm epigenetics to the embryonic development [9]. Aberrant sperm DNA methylation has been suggested to be associated with decreased male fertility, low embryo quality, and susceptibility to offspring disorders [10,11,12,13]. For instance, Kenneth et al. (2015) reported that sperm methylation patterns of infertile men were significantly different from those of fertile ones [11]. Milekic et al. (2015) found that alterations of sperm DNA methylation during aging were related to abnormal behaviors of offspring, and can be transmitted to offspring [14]. Lambert et al. (2018) observed that DNA methylation levels in bull sperm went through a change during puberty and then became stable after the age of 1 year [15]. More recently, Donkin et al. (2018) reported that alterations of the phenotypes of offspring are partially due to environmentally-driven sperm-borne methylation [16]. However, due to the lack of accurate large-scale male fertility records (i.e., limited mating times), few comprehensive studies have investigated the relationship of sperm DNA methylation with male fertility traits in mammals. DNA methylation has been studied in multiple contexts, such as global CpGs, CpG islands (CGIs), hypomethylated regions (HMRs), partially methylated domains (PMDs), etc [17,18]. For example, HMRs often occur both inside and outside of CGIs, functioning as cell type-specific enhancers. PMDs are large domains of DNA (often greater than 100 kb) that have lower levels (~40%) of DNA methylation [19,20,21,22,23,24]. They are often associated with inaccessible chromatin and inactive histone marks.

Highly-reliable male fertility phenotypes of large-scale Holstein bulls in U.S. provide a valuable source for understanding the genetics of male fertility in mammals, which are estimated based on the performance of both themselves and hundreds of thousands of their offspring. We recently conducted genome-wide association studies (GWAS) for 35 complex phenotypes with a sample size of 27,214 Holstein bulls using imputed sequence variants (~3 millions) to explore their underlying genetic architecture (e.g., causal variants and their effects), including 12 fertility, 17 body type, and 6 production traits [25]. Here, we sequenced ten Holstein cattle sperm using the whole-genome bisulfite sequencing (WGBS) technology, including six samples collected from six age-matched individuals, among which three had extreme high sire conception rate (SCR) and the remaining ones had extreme low SCR, and four samples collected from two individuals at young and old ages. We aimed to determine the alterations of sperm DNA methylation that were associated with aging and SCR first, and then to explore overlaps of these methylation alterations with GWAS signals of complex phenotypes, particularly in male fertility traits. We hypothesized that studying DNA methylome in sperm could contribute to the understanding of these complex traits. Our study revealed that the sperm DNA methylome might be associated with causal variants underlying male fertility in cattle, and thus could provide new hypotheses about the genetic and epigenetic basis of male fertility for mammals, including humans.

## 2. Results

### 2.1. Analyses of Global Methylome in Cattle Sperm

**Figure 1:**
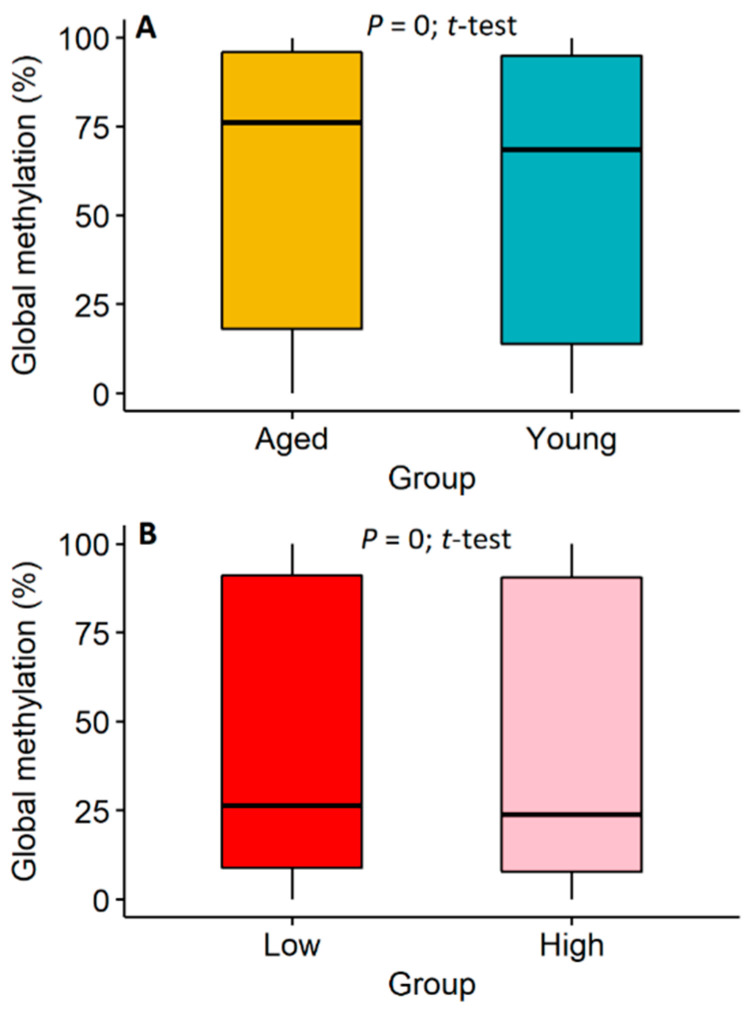
Global alterations of sperm methylation during aging and between animals with low and high sire-conception rates (SCR). (**A**) Global alterations of sperm methylation between aged and young animals. (**B**) Global alterations of sperm methylation between animals with low and high sire conception rate (SCR). The *p*-values are calculated, using the *t*-test, across all the common CpG loci of methylation information between groups.

In total, our WGBS data for all ten samples (Table S1) had an average mapping rate of 71.47% with a methylation level of approximately 75% across all CpG loci in the genome. Averages of 8,265,108 and 31,326,325 GpG loci on both strands were covered by at least 10 and 5 reads, respectively, across all ten samples. Methylation rates in CHG and CHH contexts were approximately 0.69% and 0.67%, respectively. Details of the raw data profiling are provided in Table S2. In general, we found that overall methylation levels of aged animals were significantly (*p* = 0, *t*-test) higher than those of young animals, revealing that aging could lead to a significant gain of DNA methylation in sperm globally (Figure 1A). We also found that overall methylation levels of low-SCR animals were slightly but significantly (*p* = 0, *t*-test) higher than those of high-SCR animals (Figure 1B). Together, these findings were generally consistent with previous observations in humans [26,27], which suggested that aging might lead to increased DNA methylation level and decreased male fertility.

### 2.2. Alterations in Sperm Hypomethylated Regions (HMRs) and Partially Methylated Domains (PMDs)

**Figure 2:**
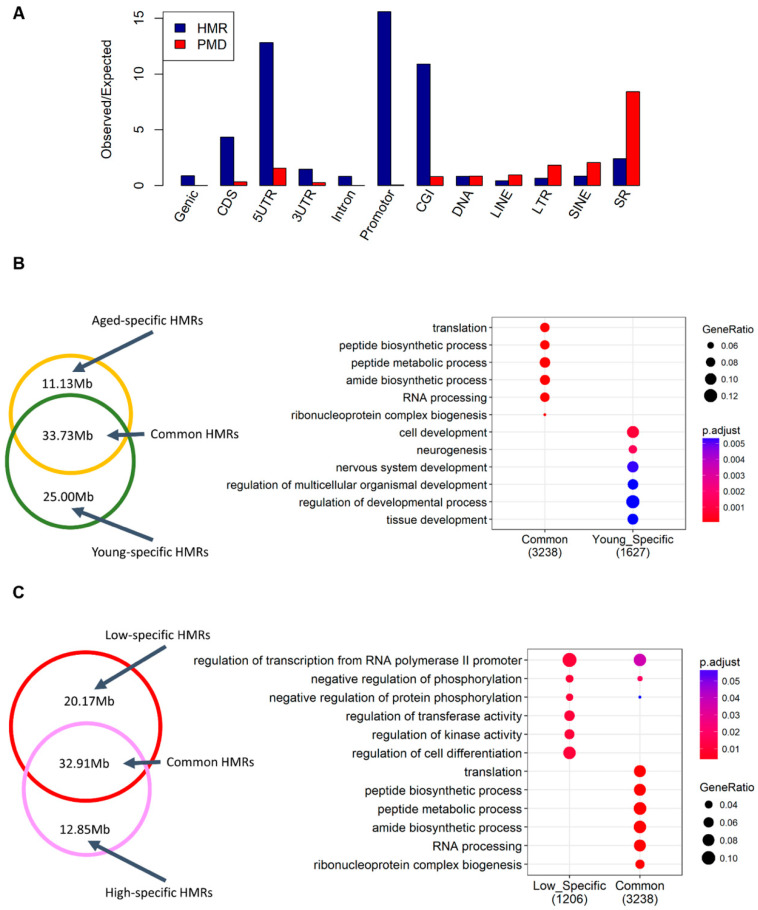
Characteristics of hypomethylated regions (HMRs) and partially methylated domains (PMDs) in sperm. (**A**) The enrichment of HMRs and PMDs across different genomic elements. (**B**) The Venn diagram (**left**) shows the comparison of HMRs at aged vs. young, while the dot plot (**right**) shows the functional enrichment results for genes whose promoters overlapped with corresponding HMR groups. (**C**) The Venn diagram (left) shows the comparison of HMRs at low-SCR vs. high-SCR, while the dot plot (right) shows the functional enrichment results for genes whose promoters overlapped with corresponding HMR groups.

We subsequently explored the hypomethylated regions (HMRs) and partially methylated domains (PMDs) in sperm as previously described [17], due to their potential roles in the gene and chromatin activation and regulation [28,29,30]. Totally, we detected 128,842 (covering ~1.69% of the cattle genome), 154,019 (~2.22%), 124,505 (~2.00%) and 127,751 (~1.73%) HMRs for aged, young, low-SCR, and high-SCR animals, respectively. We also detected 78 (~1.48%), 90 (~1.85%), 95 (~1.85%), and 98 (~2.09%) PMDs for aged, young, low-SCR, and high-SCR animals, respectively. HMRs and PMDs overlapped with many genomic features. HMRs highly intersected promoters and CGIs, while PMDs were enriched at repeated elements, such as SINE and sample repeats (SR) (Figure 2A).

*HMRs*: Most HMRs were shared among aged and young animals, and genes whose promoters intersected these shared HMRs were significantly (FDR < 0.05) engaged in translation, peptide biosynthetic, and metabolic processes (Figure 2B). Of note were genes whose promoters intersected young-specific HMRs, which were significantly involved in cell and tissue development (Figure 2B), while no significant biological processes were detected for genes with promoters which intersected aged-specific HMRs. For comparison between low-SCR and high-SCR animals, we found that genes whose promoters intersected the shared HMRs were also significantly engaged in translation, peptide biosynthetic, and metabolic processes, while genes with promoters intersected low-specific HMRs were significantly involved in regulation of transcription and phosphorylation, and kinase activity.

**Figure 3:**
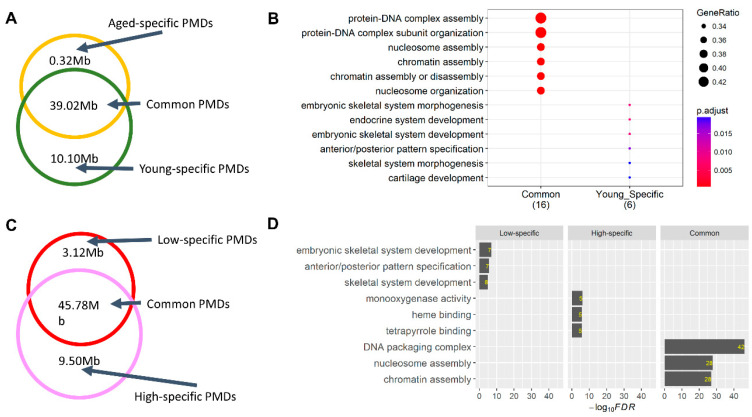
Comparisons of partially methylated domains (PMDs) in sperm. (**A**) Comparisons of PMDs between aged vs. young samples. (**B**) Functional enrichment results for genes located in common and young-specific PMDs. No significant enrichment of biological processes was found for genes located in aged-specific PMDs. (**C**) Comparisons of PMDs between low-SCR vs. high-SCR samples. (**D**) Functional enrichment analysis for genes located in low-specific, common, and high-specific PMDs. Values within the bar plot are the number of genes that are located in both the corresponding PMD group and biological process.

*PMDs*: Most PMDs were shared among animals in both comparisons (Figure 3A,C). Genes located in the shared PMDs were significantly engaged in chromatin and nucleosome assembly, consistent in both aged vs. young and low-SCR and high-SCR comparisons. Interestingly, genes located in young-specific PMDs and low-specific PMDs were highly enriched in the embryonic development (Figure 3B,D), including multiple HOX genes, microRNAs, and ZNF genes. Of note, young-specific PMDs mainly harbored HOXA genes, such as *HOXA2* and *HOXA3* (Table S3), while low-SCR-specific PMDs mainly contained HOXC and HOXD genes, such as *HOXC5* and *HOXD9* (Table S4). Genes located in high-SCR-specific PMDs significantly participated in monooxygenase activity and heme binding (Figure 3D). These findings were in line with a hypothesis that alterations of certain sperm PMDs and HMRs could be involved in male fertility through the regulation of embryonic development, despite of the first wave of DNA methylation reprogramming during the early embryogenesis.

### 2.3. Genome-Wide Differentially Methylated Regions (DMRs)

To further illustrate the associations of methylation alterations with male fertility, we subsequently performed genome-wide detection of DMRs in the comparisons of aged vs. young and low-SCR vs. high-SCR, respectively, and analyzed their associations with male fertility traits in cattle by integrating large-scale GWAS results of 35 complex traits.

**Figure 4:**
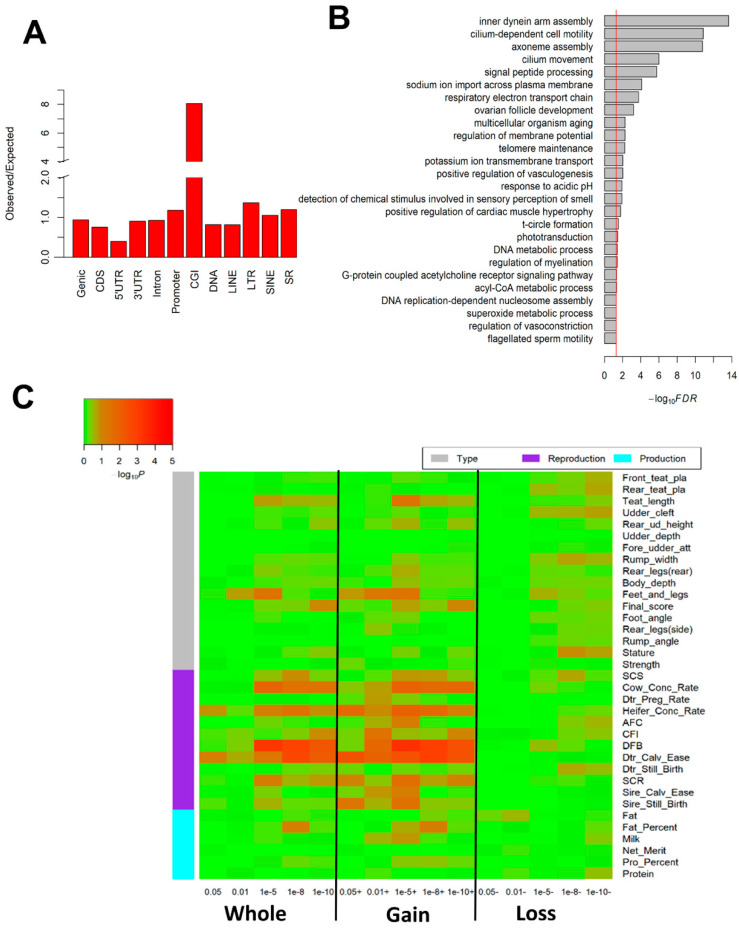
Aging-induced sperm DNA methylation alterations impact male fertility in cattle. (**A**) The enrichment of age-induced differentially methylated regions (DMRs) across genomic elements. (**B**) Significantly enriched Gene Ontology (GO) terms for age-induced DMRs. (**C**) GWAS signal enrichment for age-induced DMRs that were defined by different *q* (SLIM adjusted *p*) and difference in methylation (diff.meth) cutoffs: **Whole** represents DMRs with the absolute value of diff.meth > 5% and *q* < 0.05, 0.01, 1 × 10^−5^, 1 × 10^−8^ and 1 × 10^−10^, respectively; **Gain** represents DMRs with diff.meth > 5% in the comparison of aged vs. young, and *q* < 0.05, 0.01, 1 × 10^−5^, 1 × 10^−8^ and 1 × 10^−10^, respectively; **Loss** represents diff.meth < −5% in the comparison of aged vs. young, and *q* < 0.05, 0.01, 1 × 10^−5^, 1 × 10^−8^ and 1 × 10^−10^, respectively.

*Age-induced DMRs*: We detected a total of 16,565 DMRs (*q* < 0.01 and difference in methylation >5%), which highly intersected CGIs (Figure 4A). DMR-set enrichment analysis revealed that age-induced DMRs were significantly (FDR < 0.05) enriched in aging- and fertility-related biological processes, including multicellular organism aging, telomere maintenance, inner dynein arm assembly, cilium-dependent cell motility, and flagellated sperm motility (Figure 4B). Similar results held for DMC (i.e., differentially methylated cytosine) (Table S5). GWAS signal enrichment analysis revealed that age-induced DMRs based on five different *q*-value cut-offs (i.e., 0.05, 0.01, 1 × 10^−5^, 1 × 10^−8^, and l × 10^−10^) were significantly and selectively enriched for fertility-related traits. Of specific note were DMRs that gained methylation during aging, which showed a significantly (*p* = 2.8 × 10^−10^; Wilcoxon test) higher enrichment than those that lost methylation (Figure 4C).

**Figure 5:**
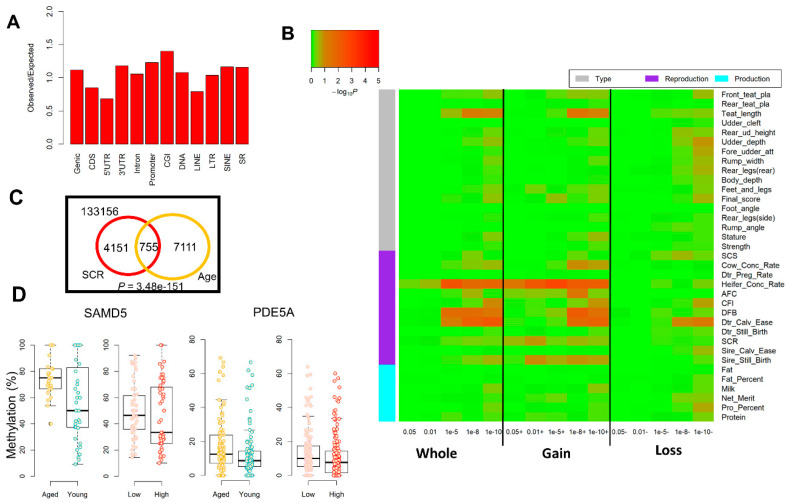
Sperm DNA methylation alterations associated with the sire-conception rate (SCR). (**A**) Enrichment of SCR-associated differentially methylated regions (DMRs) across genomic elements. (**B**) GWAS signal enrichment for SCR-associated DMRs that were defined by different *q* (SLIM adjusted *p*) and difference in methylation (diff.meth) cutoffs: **Whole** represents DMRs with the absolute value of diff.meth > 5% and *q* < 0.05, 0.01, 1 × 10^−5^, 1 × 10^−8^ and 1 × 10^−10^, respectively; **Gain** represents DMRs with diff.meth > 5% in the comparison of low vs. high, and *q* < 0.05, 0.01, 1 × 10^−5^, 1 × 10^−8^ and 1 × 10^−10^, respectively; **Loss** represents DMRs with diff.meth <−5% in the comparison of low vs. high, and *q* < 0.05, 0.01, 1 × 10^−5^, 1 × 10^−8^ and 1 × 10^−10^, respectively. (**C**) The intersection of SCR-associated DMRs and age-induced DMRs, and the *p*-value is calculated using the Fisher exact test. (**D**) DMRs in *SAMD5* and *PDE5A* genes gain methylation in aged and low sir-conception-rate (SCR) groups compared to young and high SCR groups, respectively.

*SCR-associated DMRs*: In total, we detected 11,663 SCR-associated DMRs (*q* < 0.01 and difference in methylation >5%) by analyzing the age-matched animals with extreme phenotypes, which preferred to intersect with CGIs similar to age-induced DMRs (Figure 5A). DMR-set enrichment analysis revealed that SCR-associated DMRs were significantly enriched in two sperm-activity relevant biological processes, i.e., inner dynein arm assembly and cilium-dependent cell motility (Table S6), and both of them were shared with age-induced DMRs. Similar to age-induced DMRs, we also found that SCR-associated DMRs were significantly and selectively enriched for GWAS signals of reproductive traits, and DMRs that gained methylation in low-SCR group had a significantly higher enrichment than DMRs that lost methylation (Figure 5B).

We also compared age-induced DMRs with SCR-associated DMRs. Among a total of 145,173 commonly tested tiles, we found 4906 and 7866 SCR-associated DMRs and age-induced DMRs, respectively. Among those DMRs, 755 were shared by SCR and age groups, which was more often than expected (*p* = 3.48 × 10^−151^; Fisher exact test) (Figure 5C). Out of the 775 shared DMRs, 458 gained methylation in both aged and low-SCR groups, which were associated with 66 genes (i.e., overlapped with the promoter and gene body) (Table S7). Out of the 66 genes, 16 harbored suggestive significant (*p* < 1 × 10^−5^) SNPs of reproductive traits in cattle (Table 1), suggesting their potential roles in male fertility. We showed DMRs within *SAMD5* and *PDE5A* genes as examples in Figure 5D, which gained methylation during aging and in animals with low-SCR compared to those with high-SCR, and contained suggestively significant GWAS hits of SCR. *SAMD5* is the target of *EGR4*, which has a well-established role in male infertility but no female infertility due to the arrested spermatogenesis [31]. The *PDE5A* affects erectile dysfunction, causing the male sub-fertility [32,33]. Our results provided additional evidence that aging might have an impact on male fertility, potentially through mediating the sperm DNA methylation.

## 3. Discussion

In this study, we for the first time explored the associations of sperm methylation with male fertility traits in cattle by overlapping signals of a large-scale GWAS of 35 complex phenotypes with sperm DNA methylomes. We demonstrated that multiple sperm methylation features of biological importance were associated with aging and SCR, such as HMR and PMD. We speculated that genes in the young-specific HMRs and PMDs (e.g., HOX gene clusters and microRNAs) could play significant roles in embryonic development, similar for genes in the low-SCR-specific PMDs. These findings were consistent with previous human studies in which sperm methylation patterns were essential for embryonic development [9,10,12,14,16]. For instance, promoters of development genes (e.g., microRNA clusters and HOX gene clusters) were generally hypomethylated in sperm, and the retained nucleosomes in sperm were enriched at these genes [9]. Although most of DNA methylation were reprogramed during early embryonic development and sperm maturing, some marks are maintained, such as imprinted genes, as a prime example. Additionally, Wang et al. (2014) reported that the methylation levels of a fraction (~6.8%) of GpG sits in mouse sperm retained across embryonic developmental stages [34]. More recently, Aston et al. (2015) and Jenkins et al. (2016) showed that the sperm DNA methylation state influences male fertility and embryo quality in humans [11,13], consistent with our findings in cattle that DNA methylation alterations (i.e., DMRs) associated with aging and SCR were significantly and selectively enriched with GWAS signals of male fertility traits. Additionally, Jenkins et al. (2014) showed that sperm DNA methylation alterations in aged fathers might contribute to the increased incidence of neuropsychiatric and other diseases in their offspring [10]. Atsem et al. (2016) also demonstrated that the methylation changes of *FOXK1* and *KCNA7* in sperm induced by aging can be transmitted to the next generation [12]. These are consistent with our findings that several human-homologous neurodevelopmental genes experienced methylation alterations during aging in cattle, such as *FOXP1* and *FOXN1* (Table 1. Together, we speculated that the sperm DNA methylome not only can facilitate mature gamete function, but also can guide the early embryogenesis and even influence the later life and offspring.

Alterations in epigenome have been proposed to capture and mediate the effects of genetic and environmental factors on complex phenotypes [19]. Mounting evidence supports that the comprehensive functional annotations play a central role in understanding gene expression, cellular differentiation, and complex phenotypes. For instance, the Roadmap Epigenomics Consortium (2015) characterized the epigenome maps for 127 somatic tissues and cell types in humans, and demonstrated that genomic variants of complex traits and diseases were enriched in epigenetic marks of trait-relevant tissues [35]. Here, we provided new insights into the genetic and biological basis underlying male fertility in cattle by overlapping sperm methylation information with GWAS signals. For example, age- and SCR-associated DMRs in sperm were significantly and selectively enriched for GWAS signals of fertility traits rather than those of production and body conformation traits.

Limitations and future directions: It is noted that our sample size of sperm DNA methylome was relatively small and our threshold used for methylation difference (5%) was relatively low. However, Teschendorff et al. (2018) reviewed that DNA methylation alterations associated with complex phenotypes are often much small (~5%) [36], such as many smoking-associated DMRs detected in whole blood [37], which were not like cancer DMRs (~30%) [36]. Additionally, we tried the 20% threshold and/or applied different corrected *p*-values for multiple tests (i.e., 0.05, 0.01, 1 × 10^−5^, 1 × 10^−8^ and 1 × 10^−10^). All their GWAS enrichment results were similar. We detected 16 candidate genes for male fertility, and 4 of them were directly associated with SCR, while the others were associated with other male fertility traits (Table 1). This is mainly because of the low heritability of SCR, and SCR was genetically correlated with other fertility traits that tended to have higher heritability [25]. Technical validation of these results, using independent technologies and more samples, is warranted for a future investigation. Additionally, further functional studies are required to estimate the rate of conservation of differential DNA methylation during embryonic development and the extent through which these preserved changes are able to influence gene expression leading to a modification of phenotypic outcomes. It could also be interesting to further explore whether mutations within DMR directly control complex traits or indirectly affect methylation state and then act on such complex traits. A large-scale epiGWAS focused on such regions can help us answer such questions. We did not consider non-CpG methylation in the present study, because we only observed a very small fraction (less than 1%) of non-CpG methylation in cattle sperm. However, it could be of interest to investigate the specific functions of non-CpG methylation in sperm and their associations with complex traits in offspring in the future study.

## 4. Materials and Methods

### 4.1. Sample Description and Whole-Genome Bisulfite Sequencing (WGBS)

No animal experiments were performed in this study, and ethics committee approval was therefore not required. We collected ten semen straws from eight healthy and representative Holstein bulls, including six samples collected from six age-matched individuals, among which three had extreme high sire conception rate (SCR) and another three with extreme low SCR, and four samples collected from another two individuals at young and old ages (Table S1). We isolated genomic DNA by using QIAamp DNA Mini Kit protocol (QIAGEN, Valencia, CA, USA), and evaluated the quality of isolated DNA by using the 2100 Bioanalyzer (Agilent Technologies, Santa Clara, CA, USA). We constructed the whole-genome bisulfite sequencing libraries using all the qualified genomic DNA (details in Reference [17]), and then sequenced using HiSeq X Ten (Illumina, San Diego, CA, USA) with a 150 bp paired-end technology.

### 4.2. Raw Data Profiling and DNA Methylation Calling

We applied FastQC v 0.11.2 (https://www.bioinformatics.babraham.ac.uk/projects/fastqc/) and Trim Galore v 0.4.0 (https://www.bioinformatics.babraham.ac.uk/projects/trim_galore/) to evaluate the raw data quality and to filter the low-quality data, respectively [17]. Generally, the adapters were removed, and reads with low quality (Q < 20) and shorter than 20 bp were removed. We aligned the filtered data (i.e., clean data) to the cattle reference genome UMD 3.1 using bowtie2 [38]. We then employed Bismark software to extract methylcytosine information [39]. Details have been described previously [17]. Averages of 8,265,108 and 31,326,325 CpG loci on both strands were covered by at least 10 and 5 reads, respectively, across all ten samples (Table S1).

### 4.3. Detection of Differentially Methylated Regions (DMRs) and Differentially Methylated Cytosines (DMCs)

We employed methylKit to conduct DMR/DMC analysis [40]. For DMR, we first tiled the whole genome with non-overlapping windows (500 bp in length), and then summarized the methylation levels on those tiles. We then applied a logistic regression model, implemented in *calculateDiffMeth* function, to detect DMRs. *p*-values were obtained by comparing the model fitness of alternative models to null models, and were adjusted to *q*-values for multiple testing using the SLIM method [41]. Since DNA methylation alterations associated with age and complex phenotypes are generally much weak [36,42], we used the absolute value of difference in methylation >5% and different *q* cutoffs (i.e., 0.05, 0.01, 1 × 10^−5^, 1 × 10^−8^ and 1 × 10^−10^) to define DMRs for the downstream analyses. We detected DMC with the same procedure. In the following analyses, we focused on DMR. The desirable reasons for analyzing DMRs instead of DMCs have been reviewed in detail [36]. Briefly, (1) DNA methylation tends to be highly correlated within approximate 500 bp due to the processivity of DNA methyltransferases [42,43], and alterations in DNA methylation that are associated with age and complex traits often exhibit such spatial correlation patterns [42], thus DMRs are more functionally important than DMCs. (2) Detecting DMR helps remove some of the spatial redundancy, thus reducing the burden for multiple testing and improving statistical robustness, particularly in the context of WGBS data with limited coverage [44,45]. 

### 4.4. PMD and HMR Identification

All the CGs with at least 5× coverage were used for PMD and HMR detection, as described by our earlier study [17]. For PMD detection, we first calculated the average methylation level for each of the nonoverlapped 20-kb windows. The windows with methylation level greater than 60% were assigned a 1 and the windows with methylation level less than 60% were assigned a 0. Then, we applied a hidden Markov model using HMM (one R package) to detect the windows assigned with continuous 0 for each sperm sample. The sperm PMDs used in this study had to meet the following criteria: Supported by at least three sperm samples and combined from at least three windows.

To identify the contiguous HMRs for sperm cells and somatic cells, we used a sliding window approach with a window size of 200 bp and extended the window by 50-bp steps until it contained less than 80% hypomethylated (methylation level <20%) CpGs. Only the HMRs with at least 5 CGs detected with more than 5 × coverage were used for analysis. The GenomicRanges package in R was used to calculate statistics of the overlapped HMRs in different tissues or sperm.

### 4.5. GWAS Summary Statistics

Details of the GWAS analysis for the 35 complex traits in cattle were described previously [25]. Briefly, we employed a linear mixed model, implemented in MMAP (https://mmap.github.io/), to conduct single-marker GWAS analysis for each complex trait with the sample size of 27,214 Holstein bulls and imputed sequence variants (~3 million SNPs). The model measured additive effects of genetic variants while accounting for the population structure and individual relatedness with a genomic relationship matrix. All the phenotypes of bulls (i.e., de-regressed breeding values) used in the current study were carefully calculated based on the performance of many offspring, while accounting for known systematic effects. Details are described in the CDCB website (https://www.uscdcb.com/what-we-do/genetic-evaluations/). We have classified the 35 complex traits into three phenotypic categories [25], including 17 body type, 12 fertility, and 6 production traits.

### 4.6. GWAS Signal Enrichment Analysis for Differentially Methylated Regions (DMRs)

We employed the following sum-based marker-set test approach to examine the enrichment of GWAS signals in genomic features (i.e., age-induced DMRs) due to the fact that the complex phenotypes being studied are highly polygenic or even omnigenic [46]. Previous studies demonstrated that this method exhibited higher or at least equal power compared to other commonly used marker-set test methods (e.g., count-based, score-based and covariance-based) in humans [47], *Drosophila melanogaster* [48] and livestock [49,50,51], particularly in the highly polygenic traits context.

(1) $$T_{sum}=\overset{m_{f}}{\underset{i=1}{\sum }}t^{2}$$

in which $m_{f}$ is the number of genetic variants within a genomic feature (e.g., age-induced DMRs), and $t^{2}$ is the square of *t* that was computed as the variant effect (*b*) divided by the corresponding standard error. Here, different elements (e.g., individual DMRs) within a genomic feature (e.g., age-induced DMR list) were often not in linkage disequilibrium (LD), as they scattered distantly or even on different chromosomes. This method investigated the genome-wide polygenic signals rather than a subset of variants that passed a certain significance threshold, like linkage disequilibrium (LD) score regression [52]. It took account for LD patterns among variants and variant-set sizes through applying the following cyclical permutation strategy, as described previously in References [47,48]. In brief, we first ranked the test statistics (i.e., $t^{2}$) for all variants according to their physical positions (i.e., $t_{1}^{2}$, $t_{2}^{2}$, ⋯ $t_{m-1}^{2}$, $t_{m}^{2}$). We randomly selected one test statistic (i.e., $t_{k}^{2}$) from this vector as the first, and then shifted the remaining test statistics to new positions, while maintaining their original orders (i.e., $t_{k}^{2}$, $t_{k+1}^{2}$,⋯ $t_{m}^{2}$,$\;t_{1}^{2}$,⋯ $t_{k-1}^{2}$). Therefore, we uncoupled associations of variants with a genomic feature while retaining the correlation patterns among test statistics of variants. We computed a new summary statistic for a particular genomic feature being analyzed according to its original chromosome position. We repeated this permutation procedure 10,000 times for each genomic feature and obtained an empirical *p*-value using one-tailed tests of the proportion of random summary statistics greater than that observed. The current marker-set test method together with multiple quantitative genomic tools are implemented in the QGG package (http://psoerensen.github.io/qgg/).

### 4.7. DMR/DMC-Set Enrichment Analysis Based on GO Database

We used Bboconductor package “org.Bt.eg.db” v. 3.3.0 (https://bioconductor.org/packages/release/data/annotation/html/org.Bt.eg.db.html) to link genes to GO terms. Here we focused on biological processes terms that comprised of at least 10 directly evidenced genes. We used the following count-based approach to test whether a GO term enriched for DMR or DMC.

(2) $$T_{count}=\;\overset{m_{f}}{\underset{i=1}{\sum }}I(p_{i}<p_{0})$$

where $m_{f}$ is the total number of 500 bp-windows being tested that overlaps with genes in a GO term, *p*_i_ is the *p*-value for the *i*th tested window, $p_{0}$ is an arbitrary chosen threshold, and I is an indicator function that takes one when *p*_i_ < *p*_0_ is satisfied, otherwise it takes zero. Here we arbitrarily used *p*_0_ = 0.01, and extended gene regions 10 kb up- and down-stream to include the regulatory regions. Under the null hypothesis (i.e., DMR or DMC are distributed randomly in the genome), we assumed that *T*_count_ follows a hypergeometric distribution: *T*_count_~Hyper(*m*, *m*_g_, *m*_f_), where m is the total number of windows being tested in the whole genome, m_g_ is the total number of DMR in the whole genome, and m_f_ is the number of windows being tested in a GO term. Here, we considered a window belonging to a GO term if the window overlapped with genes (± 10 kb up and down-stream) in the GO term. We used the same procedure for DMC-set enrichment analysis.

### 4.8. Gene-Set Functional Enrichment Analysis

We conducted functional enrichment analyses for gene lists using R package clusterProfiler [53], where a hypergeometric test, based on the current GO database, was employed. We focused on Biological Processes in the GO database. We adjusted *p*-values for multiple testing using the FDR method [54].

### 4.9. Availability of Data and Materials

The ten cattle sperm methylomes have been submitted to NCBI under GEO accession ID GSE106538 and GSE119263. All genomic annotation files are available for download from Ensembl database (https://uswest.ensembl.org/index.html). The GO annotation database can be publicly accessed (https://bioconductor.org/packages/release/data/annotation/html/org.Bt.eg.db.html).

## 5. Conclusions

By integrating sperm methylation with a large-scale GWAS of complex traits in cattle, we demonstrated that alterations in sperm DNA methylation were significantly associated with male fertility traits. Our results supported a hypothesis that genes in certain young-specific HMRs and PMDs might play significant roles in embryonic development. We detected 16 genes that gained DNA methylation in sperm during aging and in low-SCR animals, as well as harbored suggestively significant genetic variants of male fertility traits. Our current study provided new hypotheses about the genetic and biological mechanisms underlying male fertility, which will benefit other species, including humans. As functional annotations improve dramatically, such integrative analysis will become more interesting and useful in the near future to better understand the molecular mechanisms underlying diverse complex traits and diseases.

## Figures and Tables

**Table 1:** Genes (gene body and promoter) bearing both age- and SCR-associated differentially methylated regions (DMR) and suggestive significant SNPs (*p* < 1 × 10^−5^) of reproduction traits in cattle.

CHR	DMR Start	DMR End	*q* (Age)	Meth. Diff. (Age)	*q* (SCR)	Meth. Diff. (SCR)	Gene	Associated Traits	Top SNP Position	*p*-Value
**9**	85957501	85958000	1.11 × 10^−7^	31.92	1.24 × 10^−3^	14.72	*SAMD5*	SCR	85988014	4.74 × 10^−7^
**6**	7012001	7012500	1.41 × 10^−6^	5.51	9.38 × 10^−12^	5.42	*PDE5A*	SCR	6880354	6.44 × 10^−6^
**5**	117709001	117709500	4.88 × 10^−3^	8.90	5.96 × 10^−3^	7.10	*GTSE1*	SCR	117689528	6.19 × 10^−6^
								Sire_Calv_Ease	117691137	4.89 × 10^−6^
**27**	30563001	30563500	1.13 × 10^−4^	6.98	1.22 × 10^−6^	6.73	*UNC5D*	SCR	30756178	5.71 × 10^−6^
**1**	147329501	147330000	3.92 × 10^−5^	34.09	1.09 × 10^−7^	25.52	*PCBP3*	AFC	147188444	6.37 × 10^−6^
**22**	30725501	30726000	4.05 × 10^−3^	27.20	5.19 × 10^−3^	12.16	*FOXP1*	Sir_Calv_Ease	30678479	4.86 × 10^−6^
**8**	92536501	92537000	2.03 × 10^−5^	6.15	4.82 × 10^−6^	5.22	*PLPPR1*	Dtr_Still_Birth	92354263	5.14 × 10^−7^
								Sire_Still_Birth	92503882	9.70 × 10^−7^
**28**	7153001	7153500	9.68 × 10^−3^	19.49	4.03 × 10^−3^	22.46	*SLC35F3*	Sir_Still_Birth	6894934	5.02 × 10^−10^
**6**	115334001	115334500	8.21 × 10^−3^	31.99	4.58 × 10^−3^	10.88	*C1QTNF7*	Dtr_Calv_Ease	115292546	1.24 × 10^−7^
**28**	29788001	29788500	3.99 × 10^−4^	5.53	1.64 × 10^−3^	5.07	*MYOZ1*	Cow_Conc_Rate	29798384	2.92 × 10^−6^
**6**	89203501	89204000	6.86 × 10^−3^	6.41	8.49 × 10^−3^	5.38	*ADAMTS3*	CFI	89297757	9.55 × 10^−9^
								Dtr_Preg_Rate	89436001	1.04 × 10^−7^
								Dtr_Still_Birth	89227960	6.15 × 10^−7^
**9**	50054001	50054500	2.51 × 10^−3^	8.90	1.86 × 10^−3^	8.68	*ASCC3*	AFC	49838161	2.18 × 10^−6^
								Sire_Calv_Ease	49839702	5.74 × 10^−6^
**13**	26858001	26858500	8.21 × 10^−3^	20.41	2.55 × 10^−3^	8.13	*MYO3A*	Sire_Still_Birth	26887898	1.52 × 10^−6^
**19**	12575001	12575500	9.96 × 10^−11^	31.08	4.82 × 10^−6^	16.16	*BCAS3*	Heifer_Conc_Rate	12459490	1.18 × 10^−9^
								Sire_Calv_Ease	12432636	5.06 × 10^−7^
								Sire_Still_Birth	12432710	3.56 × 10^−7^
**3**	101902501	101903000	4.66 × 10^−3^	16.05	1.77 × 10^−3^	8.81	*C1orf228*	CFI	10191696	7.16 × 10^−6^
								Sire_Still_Birth	101936626	7.16 × 10^−6^
**4**	57398001	57398500	3.21 × 10^−7^	5.68	7.47 × 10^−10^	5.25	*IMMP2L*	Sire_Still_Birth	57140432	3.90 × 10^−7^

Note: SCR: Sire conception rate; Sire_Calv_Ease: Sire calving ease; AFC: Age at first calving; Dtr_Still_Birth: Daughter stillbirth; Dtr_Calv_Ease: Daughter calving ease; Cow_Conc_Rate: Cow conception rate; CFI: Interval from calving to first insemination; Dtr_Preg_Rate: Daughter pregnancy rate; Sire_Calv_Ease: Sire calving ease; Heifer_Conc_Rate: Heifer conception rate.

## Supplementary Materials

The following are available online at https://www.mdpi.com/2075-4655/3/2/10/s1: Table S1: Summary information of the 10 sperm samples studied. Table S2: Summary statistics of the raw data mapping for the 10 sperm methylomes. Table S3: Genes located in young-age-specific partially methylated domain (PMD) regions. Table S4: Genes located in low-SCR-specific partially methylated domain (PMD) regions. Table S5: (A) The enrichment analysis for age-induced differentially methylated regions (DMRs); (B) The enrichment analysis for age-induced differentially methylated cytosines (DMCs). Table S6: The enrichment analysis for SCR-associated differentially methylated regions (DMRs). Table S7: Summary of the shared age-induced DMRs and SCR-associated DMRs and their associated genes.

## Abbreviations

HMR	Hypomethylated regions
PMD	Partially methylated domains
GWAS	Genome-wide association study
SCR	Sire-conception-rate
DMR	Differentially methylated region
DMC	Differentially methylated cytosine
GO	Gene Ontology
CGI	CpG islands
